# Phytosociological Study, Diversity and Conservation Status of the Cloud Forest in the Dominican Republic

**DOI:** 10.3390/plants9060741

**Published:** 2020-06-12

**Authors:** Ana Cano Ortiz, Carmelo Maria Musarella, Carlos José Pinto Gomes, Ricardo Quinto Canas, José Carlos Piñar Fuentes, Eusebio Cano

**Affiliations:** 1 Department of Animal and Plant Biology and Ecology, Section of Botany, University of Jaén, 23071 Jaén, Spain; anacanor@hotmail.com (A.C.O.); jpinar@ujaen.es (J.C.P.F.); ecano@ujaen.es (E.C.); 2 Department of AGRARIA, Mediterranean University of Reggio Calabria, Loc. Feo di Vito snc, 89122 Reggio Calabria, Italy; 3 Department of Landscape, Environment and Planning, School of Science and Technology, MED - Mediterranean Institute for Agriculture, Environment and Development, University of Évora, 7000-671 Évora, Portugal; cpgomes@uevora.pt; 4 Faculty of Sciences and Technology, University of Algarve, Campus de Gambelas, 8005-139 Faro, Portugal; rjcanas@ualg.pt; 5 Centre of Marine Sciences (CCMAR), University of Algarve, Campus de Gambelas, 8005-139 Faro, Portugal

**Keywords:** floristic diversity, vegetation, phytosociology, cluster analysis, America, Cuba

## Abstract

The study of the forest in rainy environments of the Dominican Republic reveals the presence of four types of vegetation formations, clearly differentiated from each other in terms of their floristic and biogeographical composition, and also significantly different from the rainforests of Cuba. This leads us to propose two new alliances and four plant associations located in northern mountain areas exposed to moisture-laden winds from the Atlantic: All. *Rondeletio ochraceae*-*Clusion roseae* (Ass. *Cyatheo furfuracei*-*Prestoetum motanae*; Ass. *Ormosio krugii*-*Prestoetum montanae*); and All. *Rondeletio ochraceae*-*Didymopanion tremuli* (Ass. *Hyeronimo montanae*-*Magnolietum pallescentis*; *Hyeronimo dominguensis*-*Magnolietum hamorii*). We pay special attention to the description of cloud forest types, since they have a high rate of endemic species, and therefore there are endemic habitats, which need special protective actions. Therefore, we apply the Shannon diversity index to characteristic, companion, non-endemic, and endemic species. As result, the association *Ormosio krugii*-*Prestoetum montanae* has a Shannon_T = 2.4 and a value of Shannon_E = 0, whereas the other 3 associations have a better conservation status with Shannon values in all cases > 0: This is due to a worse conservation status of the Eastern Cordillera, in comparison with the Central Cordillera and Sierra de Bhaoruco. Due to human activity, some areas are very poorly conserved, as evidenced by the diversity index and the presence of endemic tree and plant elements. The worst conserved in terms of the relationship between characteristic plants vegetation (cloud forest) in areas with high rainfall are in the Dominican Republic, along with its floristic diversity and state of conservation. This study has made it possible to significantly increase the botanical knowledge of this important habitat.

## 1. Introduction

The territory of the Dominican Republic (DR), with an extension of 48,198 km^2^ including the small adjacent islands, accounts for over two thirds of the territory of Hispaniola, an island located between parallels 17–19° N in the group of the Greater Antilles. Most previous botanical studies have concentrated predominantly on the flora, for example the work of García et al. [1] in the Sierra de Bahoruco, and highlight the abundant rainfall of up to 4000 mm and the very high rate of endemic species. There are also other studies by several authors on the cloud forest in the Cordillera Central, Septentrional, and Oriental ranges [2,3,4,5,6,7,8,9,10,11,12,13,14,15,16,17,18]. All these works, together with previous studies carried out by ourselves [19,20,21,22,23,24,25,26,27,28,29,30], have enabled us to undertake the present work. All the aforementioned studies focus attention on the knowledge of the flora, with only passing references to the vegetation. There are studies of this type in the neighboring islands such as Cuba [31,32] giving similar physiognomic aspects between the two islands, but with high floristic differences. Consequently, our objective is to discern whether the existing vegetation on both islands is the same or different and, secondly, to study the cloud forest in the Dominican Republic applying the phytosociological method. Not having phytosociological studies on the cloud forest, we have only been able to use some floristic publications, and some works on vegetation, but of physiognomic type, in which the distribution of the species is revealed. These works, which together with those of ours in which we made the distribution of more than 1500 endemic species, have helped us to tackle this work of the cloud forest. Once the plant communities were described, we completed an analysis of species diversity. For this, we used the Shannon index to the different groups of species of each phytosociological table (characteristics, companions, non-endemics, and endemics), to see the state of conservation.

So, the main aim of this work is to determine the forest vegetation (cloud forest) in areas with high rainfall in the Dominican Republic, along with its floristic diversity and conservation status.

## 2. Results

**Figure 1:**
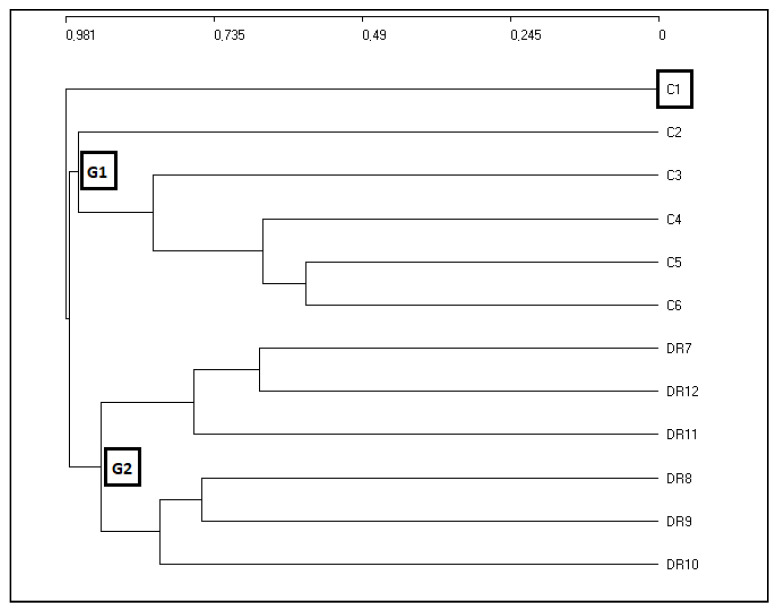
Jaccard distance cluster. Cluster analysis for the associations of Cuba and the Dominican Republic (DR). The six communities described in Cuba by [31,32,33,34,35,36,37] are separated into the community C1 and group G1 (C2, C3, C4, C5, C6). G2 includes 32 of our own relevés and those one of [9,10] from the Dominican Republic (DR) (DR7, DR8, DR9, DR10, DR11, DR12).

The results of the analysis of Jaccard distances (Figure 1), applied to six plant communities in Cuba and six in the DR, show that the six communities described in Cuba by [31,32,33,34,35,36,37] can be separated into the community C1 and the group G_1_ (C2, C3, C4, C5, C6). C1 is differentiated from the rest in terms of its floristic, structural, and ecological composition, as this is a pinewood of *Pinus maestrensis* Bise growing in rainy environments but on highly oligotrophic soils, in common with the other communities in group G_1_, which is floristically significantly different from group G_2_. There are very significant floristic differences between Cuba and the DR which can be observed analyzing Table 1 and Table 2, with 173 species present in the samplings in the DR but not in Cuba, whereas the samplings in Cuba reveal 139 plants that are absent from the DR. Establishing the floristic differentiation between both islands is essential for current phytosociological studies. In group G_2,_ which includes 32 of our own relevés and those one of [9,10] (DR7, DR8, DR9, DR10, DR11, DR12), the communities can be seen to form a group for the DR representing different types of forests; these formations are a series of plant communities in very rainy environments in the Dominican Republic (DR) located in the Sierra de Bahoruco and the Cordillera Central and Oriental ranges, with rainfall of over 2000 mm. Group G_2_ is broken down into two subgroups of plant communities DR7-DR11-DR12 and DR8-DR9-DR10, as the first three correspond to areas with acid substrates and rainy environments in the Cordillera Central range, whereas the second subgroup contains communities growing on different kinds of substrates and in hyper-humid environments. We therefore focused on the analysis of 17 of our own samplings to which we apply a Euclidean distance cluster analysis and an ordination analysis (DCA), both of which perfectly separate the sampling groups. We carried out this analysis to establish the different forests groups, and then to establish the phytosociological tables.

### 2.1. Phytosociological Study

**Figure 2:**
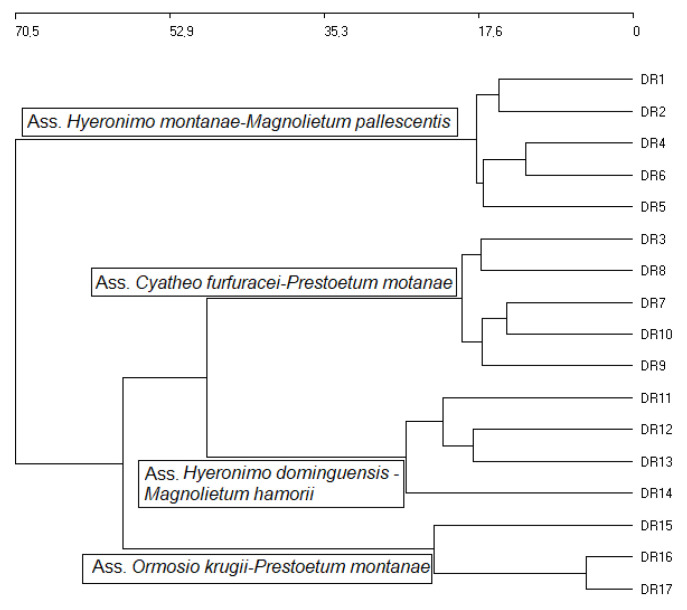
Cluster analysis for the Dominican Republic (DR) relevés. Euclidean distance using Ward’s method separating the four associations (Ass.) found in DR relevés.

**Figure 3:**
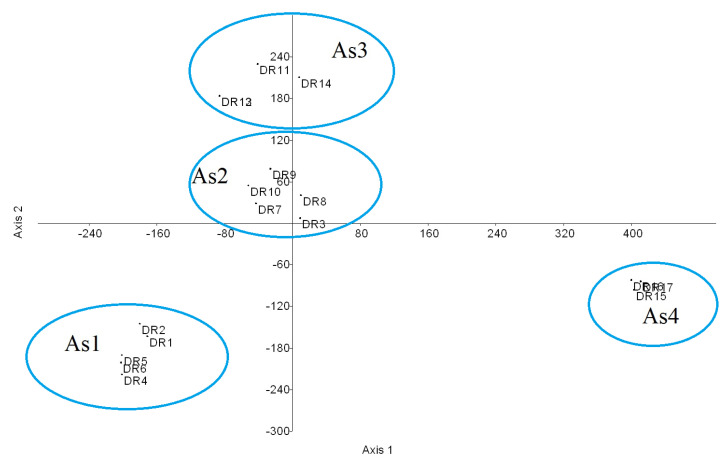
Detrended correspondence analysis (DCA). DCA analysis confirming the separation of the four associations (As1 *Hyeronimo montanae*-*Magnolietum pallescentis*; As2 *Cyatheo furfuracei*-*Prestoetum montanae*; As3 *Hyeronimo dominguensis*-*Magnolietum hamorii*; As4 *Ormosio krugii*-*Prestoetum montanae*) found in Dominican Republic (DR) relevés.

**Figure 4:**
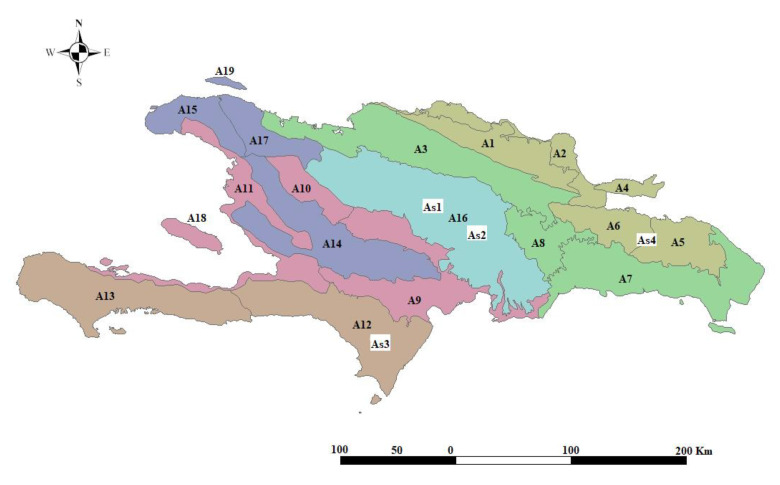
Biogeographical distribution of the associations in the study. As1: *Hyeronimo montanae*-*Magnolietum pallescentis* (A16: central district). As2: *Cyatheo furfuracei*-*Prestoetum montanae* (A16: central district). As3: *Hyeronimo dominguensis*-*Magnolietum hamorii* (A12: Bahoruco district). As4: *Ormosio krugii*-*Prestoetum montanae* (A5: eastern district) (from Cano–Ortiz et al., modified [28]).

The statistical analysis of the samplings from the DR reveals the existence of four forest plant associations (Figure 2): As1 *Hyeronimo montanae*-*Magnolietum pallescentis nova hoc loco* (Appendix A, Table A1, rel. DR1, DR2, DR4, DR5, DR6; *typus* rel. DR4), growing at altitudes of between 1300 and 1500 m on siliceous substrates in the Cordillera Central range (central biogeographical district), and in rainy environments with a humid ombrotype and a mesotropical thermotype [16,23,38,39]. These forests contact in hyper-humid areas with forests of *Prestoea montana* (Grah.) Nichol, and have a high floristic diversity with 21 trees, eight climbing species, and five epiphytes, and a high rate of endemisms (14 species); at higher altitudes, above the sea of clouds, the cloud forest of *Magnolia pallescens* contacts the pine forest of *Pinus occidentalis*, association *Dendropemom phycnophylli*-*Pinetum occidentalis* [22]. As2 *Cyatheo furfuracei*-*Prestoetum montanae nova hoc loco* (Appendix A, Table A2, rel. DR3, DR7, DR8, DR9, DR10; *typus* rel. DR3) is a plant community dominated by *Prestoea montana*, always found in hyper-humid environments, generally in very rainy and shady gorges, contacting with the previous association towards areas that are somewhat less rainy and more exposed to sun and wind. It also has a high diversity, with 40 tree and 25 epiphyte species. Due to the catenal contact between both associations, As1 and As2 have common species: therefore, they are statistically close (Figure 3). Indeed, the total diversity of the 4 associations is shown in the H’ index values, as well as in the analysis of the phytosociological tables. As3 *Hyeronimo dominguensis*-*Magnolietum hamorii nova hoc loco* (Appendix A, Table A3, rel. DR11, DR12, DR13, DR14; *typus* rel. DR11) represents forests of *Magnolia* in the Sierra de Bahoruco, which develop on calcareous substrates in humid environments at altitudes of around 1200–1300 m in a humid ombrotype and a mesotropical thermotype, with a high number of tree (25) and epiphyte (14) species; in this case the cloud forest connects with the pine forest belonging to the *Cocotrino scopari*-*Pinetum occidentalis* association. As4 *Ormosio krugii*-*Prestoetum montanae nova hoc loco* (Appendix A, Table A4 rel. DR15, DR16, DR17; *typus* rel. DR16), an association characterized by a high diversity of trees (27 species), and a lower number of endemic species than the previous associations. The four associations present a clear floristic and biogeographical differentiation (Figure 4) [17,18,40]. The biogeographic strength and the high floristic and ecological differentiation allow us to establish the four plant associations, despite not having a greater number of inventories. However, these associations present a high number of endemisms, which allows us to treat them as endemic habitats of interest for conservation. In the synthetic analysis (Table 3) the great floristic differentiation between the 4 plant communities can be seen, with the floristic differences among them being 25.7% (As1), 40.6% (As2), 20.7% (As3), and 36.6% (As4): these floristic differences between the 4 associations have been selected from the synthetic table.

The four associations described are included in the phytosociological classes *Cyrillo*-*Weinmannietea pinntae* Borhidi 1996 and *Ocoteo*-*Cyrilletea rceniflorae* Borhidi 1996. Due to the high floristic and biogeographical differentiation between Hispaniola and Cuba (Table 1 and Table 2), these associations cannot be included in any of the alliances described for the island of Cuba. We therefore propose two new alliances: All. *Rondeletio ochraceae*-*Clusion roseae*, in which the alliance species are *Rondeletia ochracea, Turpinia occidentalis, Clusia rosea, Mikania cordifolia, Alchornea latifolia*, and *Cyatheo furfuracei*-*Prestoetum motanae* as the type association; and all. *Rondeletio ochraceae*-*Didymopanion tremuli*, with the species *Rondeletia ochracea, Didymopanax tremulus, Psychotria guadalupensis,* and *Hyeronimo montanae*-*Magnolietum pallescentis* as the type association.

### 2.2. Conservation Status of the Associations

The analysis of the floristic diversity of the relevés shows a predominance of Shannon_T diversity (total diversity) over the diversity of non-endemic and endemic species, except in the samplings DR15, DR16, and DR17: They have a relative coincidence between Shannon_T and Shannon_Ne due to the low rate of endemic species, with only two species: *Bactris plumeriana* and *Clidemia umbellata*.

The diversity rate for characteristic species (Shannon_Ca) tends to be high compared to companion species (Shannon_Co), except in DR3, which has a value of Shannon_Co = 1.099 (Table 4).

**Figure 5:**
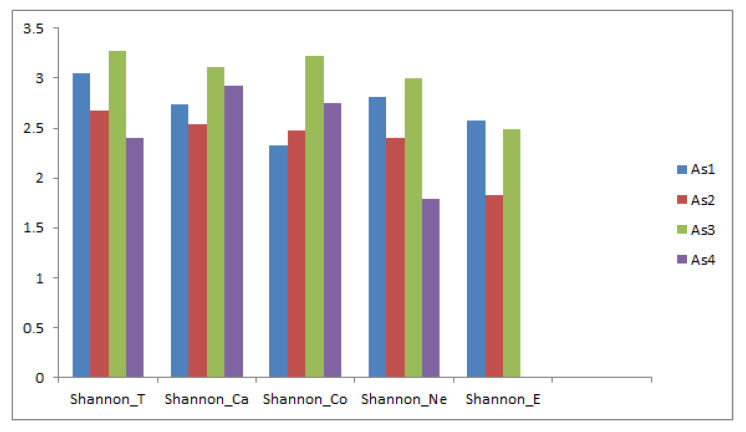
Shannon diversity values (T, Ca, Co, Ne, E) (Shannon_T = total diversity; Shannon_Ca = characteristic community species diversity; Shannon_Co = companion community species diversity; Shannon_Ne = non-endemic species diversity; Shannon_E = endemic species diversity). As1: *Hyeronimo montanae*-*Magnolietum pallescentis*. As2: *Cyatheo furfuracei*-*Prestoetum motanae*. As3: *Hyeronimo dominguensis*-*Magnolietum hamorii*. As4: *Ormosio krugii*-*Prestoetum montanae.*

In the comparative analysis of the diversity among the four associations using the average diversity values for each relevé, it can be seen that association As4 has a Shannon_E = 0 due to an almost total lack of endemic species. This association also has low values for total diversity and non-endemic species, with 44.2% trees, 22.9% shrubs, 13.1% climbing plants, and 16.3% herbs, as it appears from the study of biotypes of the phytosociological table; whereas the other associations have a greater diversity. The Shannon_Ca value is higher than the Shannon_Co in the four associations except for As3; however, the values are similar due to a tendency to ingression by companion species from neighbouring communities (Table 5, Figure 5).

## 3. Discussion

Although the diversity of the Caribbean territories has high diversity and a high rate of endemism [27], this diversity is similar to that existing in neighboring territories in South America (Colombia, Venezuela) [41,42]. There is a great difference of botanical families among the territories of Amazonia, Orinoco, and the Caribbean, but sharing families such as Buxaceae, Achatocarpaceae, and Nelumbonaceae [43], or Melastomataceae [20]: territories that present similar vegetation from the physiognomic point of view, but very different in its floristic composition [44,45,46,47].

On the island of Hispaniola, in all cases there is a high diversity of trees, among which it is particularly worth noting the endemics *Magnolia pallescens* Urb. & Ekm., *Hyeronima montana* A. Liogier, *Magnolia hamorii* Howard, *Hyeronima domingensis* Urb., *Malpighia macracantha* Ekm. & Nied., and *Bactris plumeriana* Mart. These are therefore plant communities with an endemic character that require protection measures. But it is also very important to know the dynamics of the species that characterize these plant communities, as some of them, when introduced into new geographical areas with different modalities and for different purposes, can invade the local communities, causing significant ecological damage [48]. It is evident that native species (N) have a larger distribution area, and are capable of using different ecological niches. Therefore, in the face of climatic, anthropic, and other kinds of changes, these species can expand relegating other more stenoic ones, as occurs with the endemic ones. Although all four associations are of great interest to conservation, the two best conserved associations have the highest rate of endemics, and are precisely the ones located in the Bahoruco–Hottense and central biogeographical sectors [19,21], which concurs with the previous floristic studies [1,3,11,49]. However, the areas exposed to greater environmental impact, as is the case of biogeographical sectors such as the Cordillera Oriental range which are subjected to significant human pressure, have less floristic diversity and a lower number of endemic species. No significant differences can be seen between the relevés in the Shannon diversity index, whose values range between DR3 with indexes of Sh = 2.451, and DR12 with higher values of Sh = 3.972 (Table 4); this does not imply that DR3 is poorly conserved [27], but simply that there is an almost complete predominance of the faithful species *Prestoea montana*, which has a high cover and very few companion species. However, relevé DR12 contains many individuals with low cover and a high rate of companion species. The low rate of endemisms in As4 represented by relevés DR15, DR16, and DR17 in the Cordillera Oriental range is the result of significant anthropic action owing to population density.

All these results are according to Cano Ortiz et al. [50].

### Syntaxonomical Checklist for the Cloud Forest of Hispaniola

*Cyrillo-Weinmannietea pinntae* Borhidi 1996
*Cyrillo-Weinmannietalia pinnatae* Borhdi 1996
    *Rondeletio ochraceae-Clusion roseae* Cano, Cano–Ortiz & Veloz *all. nova hoc loco*
      *Cyatheo furfuracei-Prestoetum motanae* Cano, Cano–Ortiz & Veloz *ass. nova hoc loco*
      *Ormosio krugii-Prestoetum montanae* Cano, Cano–Ortiz & Veloz *ass. nova hoc loco*
*Ocoteo-Cyrilletea racemiflorae* Borhidi 1996
*Ocoteo cuneatae-Magnolietalia cubensis* Borhidi & Muñiz in Borhidi 1996
  *Rondeletio ochraceae-Didymopanion tremuli* Cano, Cano–Ortiz & Veloz *all. nova hoc loco*
    *Hyeronimo montanae-Magnolietum pallescentis* Cano, Cano–Ortiz & Veloz *ass. nova hoc loco*
    *Hyeronimo dominguensis-Magnolietum hamorii* Cano, Cano–Ortiz & Veloz *ass. nova hoc loco*

## 4. Materials and Methods

### Study Area

The island of Hispaniola, with an area of 76,484 km^2^, and Cuba, Jamaica, and Puerto Rico are the largest islands in the Caribbean region. The geological origin of the mountains on the island dates from the Cretaceous and Oligocene–Miocene era, with the exception of the intramountain valleys formed during the Quaternary period due to the deposit of materials [51]. There is a predominance of calcareous materials with a karstic character, marbles, limestones, and Quaternary deposit materials, and a large central nucleus of siliceous materials with serpentine outcrops [20,21,22]. The island has a mountainous relief with several mountain chains such as the Oriental, Central, and Septentrional ranges, and sierras such as Bahoruco and Niebla. The steep slopes, the lack of access in certain areas, and the strong anthropic action in others pose a great difficulty for the study of these territories. The northwest-southwest orientation of the mountains and the prevailing direction of the Atlantic winds explains the existence of a permanent sea of clouds, which gives rise to high rainfall on north-northeast-facing slopes. All inventories carried out are located on slopes ranging between 15–40% and are exposed to the humid winds of the Atlantic Ocean. For the coverage in %, the dominant species of the tree vegetation have been taken into account: a study we carried out during three years of sampling (June 2005–June-July 2006 and 2007) as part of our participation in three AECI projects.

This study is focused on the humid-hyper-humid forests in the Dominican Republic (DR) on the island of Hispaniola. Vegetation samples were taken in areas of high rainfall such as the Cordillera Central and Oriental ranges and the Sierra de Bahoruco, selecting sampling plots with an area of 500–2000 m^2^. Due to the scarcity of vegetation studies, we analyzed the previous works in territories of Cuba [31,32,37]. For the dynamic-catenal landscape study we took into account the criteria of [52,53]. An Excel© table was created with 483 rows (species) × 12 columns (tables containing 67 relevés: 35 for Cuba and 32 for Dominican Republic) (Table 6).

A statistical treatment (clustering) was applied to separate the communities described for Cuba from those of Hispaniola. The flora of the 67 relevés of Cuba and Hispaniola allows us to establish a clear floristic differentiation between both islands. The statistical treatment was done by adapting the Van der Maarel conversion [54] and substituting the abundance–dominance indexes with synthetic indexes with the following equivalence: I = 3, II = 4, III = 5, IV = 6, V = 7. Once the indexes were converted, a cluster analysis was applied using the Jaccard distance, marking the distance between the associations studied. After separating the forests in the Dominican Republic (DR) from those of Cuba based on the Jaccard distance, an Excel© table was created with the vegetation relevés from the DR, and a Euclidean distance cluster analysis and a DCA were applied to obtain the different types of forests present in the DR. For this study, the statistical packages PAST (PAleontological STatistics software package for education and data analysis, v. 2.17c. Paleontological Museum, University of Oslo, Sars gate1, 0562 Oslo, Norway)© (Palaeontological Association,) and CAP3 (Community Analysis Package, PISCES Conservation Ltd. IRC House, The Square, Pennington, Lymington Hants., SO41 8GN United Kingdom)© were used.

Regarding the statistical analysis, we used 6 plant associations described in rainy environments in Cuba with a total of 35 relevés (Table 6) and 6 plant communities in the Dominican Republic with a total of 32 relevés, among which 17 were made by us. With the 12 plant communities, we made a synthetic table and, as we previously mentioned, we transformed the synthetic indices with Van der Maarel [54], with the aim of applying a cluster analysis. The Euclidean distance was not chosen since it was only used to see the similarity between two territories (Cuba and the Dominican Republic). Subsequently, an Excel© table was made exclusively with the 17 inventories carried out in the field, and we had already applied Euclidean distance, since we wanted to see the separation between the cloud forest communities, which we confirmed with a DCA analysis.

Once the 4 types of forests were separated, the phytosociological tables and the synthetic table were elaborated: this reflects the floristic difference between the 4 plant associations. For the inclusion of associations in their biogeographic units, we followed Cano–Ortiz et al. [28].

To differentiate some plant communities from others, we followed the phytosociological method of Braun–Blanquet [55] and Gehu and Rivas–Martínez [56], and we used the dynamic-chain studies by Rivas–Martínez [53]. The great floristic differentiation between the districts and biogeographical sectors established in Cano et al. [21] and Cano–Ortiz et al. [28], also with field work, is essential for the phytosociological study.

The criterion for separating syntaxa of different ranks is the distribution and ecology of the species. Obviously the most stenoic species characterize syntaxa of lower rank and the eurioics to higher syntaxonomic ranks; thus, the associations present a district or biogeographic sector distribution, while the higher rank syntaxa have a subprovince, province, superprovince, subregion, and biogeographic region distribution. For this reason, we based our investigation on previous biogeography studies carried out by us [20,21,28]. For the proposal of new syntaxa, the ICPN (International Code of Phytosociological Nomenclature) is followed [57].

The floristic study of phytosociological relevés has been verified with the work of Liogier [58] and with the herbarium specimens of the Jardín Botánico Nacional Dr. Rafael M. Moscoso of Santo Domingo (JBSD—acronym according to Thiers [59]), where the new collected specimens are also preserved.

Once the description of the 4 plant associations were made, we planned to find out the degree of conservation: for this we chose to apply the Shannon–Webeaver index or the H’ index. This measures specific diversity applying the method revealed by Cano–Ortiz et al. [29], which takes into account the relationship between the total diversity of species, the diversity of characteristics and companions. In this relationship, it is clear that each community will be better conserved, and the less its diversity of companions and the greater its diversity of characteristics, the closer it will be to total diversity. To calculate the H index, the statistical package PAST (PAleontological STatistics)© was used [60].

## 5. Conclusions

This study in the Dominican Republic reveals the existence of different types of rainforest that are clearly differentiated by their floristic, biogeographical, and bioclimatic composition. This broadleaved forest or rainforest is frequent in the Sierra de Bahoruco and the Cordillera Central, Septentrional, and Oriental ranges due to the increased rainfall in these areas caused by the impact of moisture-laden Atlantic winds. Differences in soil and biogeography have conditioned a rich and different flora. The Cordillera Central range—geologically the oldest, and with a siliceous character—is home to rainforests of *Magnolia pallescens* and forests of *Prestoea montana* (As1 and As2) in humid–hyper-humid areas; whereas the associations As3 in Bahoruco and As4 in the Cordillera Oriental range also develop in humid environments but on soil substrates. This leads us to propose four new syntaxa with the rank of association and two new alliances.

Considering the published works on Cuba, Venezuela, and Colombia already reported in the references, there are differences between these territories and the Dominican Republic. The study on the degree of conservation of the four described associations reveals a low diversity compared to other territories (Cuba, Venezuela, Colombia). Within the study territory (Dominican Republic), As4 is the worst conserved due to strong anthropic action, which has affected the rate of endemism with a Shannon_E value = 0. This association presents 27 characteristic tree species and 2 epiphytic species, of which only one is endemic, compared to 31 companion species of shrubs, herbs, and climbers.

These differences in terms of diversity and conservation status are due to the strong anthropic action that some territories present, such as the Eastern Cordillera (Dominican Republic), being this is an area dedicated mainly to livestock and agriculture, which caused deforestation.

## Figures and Tables

**Table 1:** Plant species found in Cuba [31,32] but not present in the relevés from the Dominican Republic.

*Ageratina paucibracteata* (Alain) King et Robins.	*Micropholis polita* (Griseb.) Pierre
*Alsophila aquilina* C. Chr.	*Mozartia gundlachii* (Kr. & Urb.) Urb.
*Allophyllus cristalensis* Lippold	*Myrica cacuminis* Britt and Wils.
*Ascyrum hypericoides* L.	*Myrica punctata* Griseb.
*Bactris cubensis* Burret	*Ocotea cuneata* (Griseb.) Urb.
*Buchenavia capitata* (Vahl.) Eichl.	*Oplismenus hirtellus* (L.) Beauv.
*Byrsonima biflora* Griseb.	*Ossaea muricata* (Griseb.) Wr. ex Sam.
*Byrsonima coriacea* R. Br.	*Ouratea revoluta* (Wr ex Griseb.) Engl.
*Byrsonima orientensis* Bisse	*Panicum boliviense* Hack.
*Calophyllum utile* Bisse	*Panicum glutinosum* Sw.
*Calycogonium grisebachii* Triana	*Panicum scoparium* L.
*Calycogonium lindenianum* Naud.	*Pardiaea maestrensis* Borhidi and Catassus
*Calyptrantes punctata* Griseb.	*Pera ekmanii* Urb.
*Callicarpa ferruginea* Sw.	*Phaius tankervilliae* (Banks) Blume
*Cestrum laurifolium* L’Hérit	*Pharus latifolius* L.
*Clethra cubensis* A. Rich.	*Philodendron lacerum* (Jacq.) Schott
*Clidemia strigillosa* (Sw.) DC.	*Phyllanthus pachystylus* Urb.
*Clusia minor* L.	*Phyllanthus subcarnosus* Wr ex Muell. Arg.
*Clusia tetrastigma* Vesque	*Pilocarpus racemosu*s Vahl.
*Coccocypselum* x *lanceolatum* (Urb.) Borhidi	*Pinus maestrensis* Bisse
*Coccoloba costata* Wr. Inn Sauv.	*Piper holguinianum* Trel.
*Coccoloba ekmanii* O. C. Schmidt	*Pithecellobium arboreum* (L.) Urb.
*Coccoloba retusa *Griseb.	*Platygine triandra* Borhidi
*Coussarea urbaniana* Standl.	*Pleurothalis tribuloides* (Sw.) Lindl.
*Critonia dalea* (L.) DC.	*Pleurothalis velaticaulis* Rchb.
*Cyathea araneosa* (Sw.) Domin	*Podocarpus ekmanii* Urb.
*Cyrilla nipensis* Urb.	*Polygala oblongata* (Britt.) Blake
*Chrysophyllum argenteum* Jacq.	*Polypodium phyllitidis* L.
*Dalbergaria cubensis* (Urb.) Borhidi	*Polystachya cubensis* Schltr.
*Dendropanax nervosus* (Urb et Ekm.) A. C. Sm.	*Protium cubense* (Rose) Urb.
*Dennstaedtia adiantoides* (H. & B) Moore	*Psidium balium* Urb.
*Desmodium herbaceum* (L.) Benth. & Oerst.	*Psychotria shaferi* Urb.
*Dicranopteris flexuosa* (Schrad.) Mett.	*Pteridium caudatum* (L.) Max.
*Dichaea hystricina* Rchb.	*Pteris rigida* Sw.
*Dilomilis oligophylla* (Schltr.) Summerh.	*Pytirogramma sulphurea* (Sw.) Max.
*Dinema cubincola* (Borhidi) H. Dietr.	*Rajania nipensis* Howard
*Dipholis cubensis* (Griseb.) Pierre	*Raudolfia salicifolia* Griseb.
*Ditta myricoides* Griseb.	*Rhamnidium nipense* Urb.
*Elaphoglossum firmu*m (Mett.) Urb.	*Rondeletia calophylla* Standl ex Britt.
*Eugenia rigida* Berg.	*Rondeletia naguensis* Britt and Wils
*Garrya fadyenii* Hook.	*Rubus turquinensis* Rydb.
*Gesneria pachyclada* Urb.	*Salacia nipensis* Britt.
*Gesneria viridiflora* (Dcne) Kuntze	*Sapium cubense* Britt ex Wils.
*Graffenrieda refescens* Britt. & Wils.	*Sapium erythrospermum* (Griseb.) Muell. Arg.
*Guatteria moralesii* Urb.	*Sapium jamiaicense* Sw.
*Guettarda lindeniana* A. Rich.	*Savia cuneifolia* Urb.
*Habenaria monorrhiza* (Sw.) Rchb.	*Scleria pilosissima* Britt.
*Hedyosmum grissebachii* Solms	*Schradera cubensis* Steyerm.
*Heterotrichum umbellatum* (Mill.) Urb.	*Simaruba laevis* Griseb.
*Hyeronima nipensis* Urb.	*Sloanea curatellifolia* Griseb.
*Ichnanthus mayarensis* (Wr.) Hitchc.	*Solanum cristalense* Amsh.
*Illicium cubense* A.C.Sm.	*Solanum pachyneurum* O.E. Schulz
*Jacquiniella globosa* (Jacq.) Schlechter	*Solanum virgatum* Lam.
*Lasiacis sloanei* (L.) Hitchc.	*Solonia reflexa* Urb.
*Leucocroton wrightii* Griseb.	*Stelis ophioglossoides* (Jacq.) Sw.
*Litachne pauciflora* (Swartz) P. Beauverd	*Tabebuia hypoleuca* Griseb.
*Lobelia assurgens* L.	*Talauna minor* Urb. subsp. *oblongifolia* (León) Borhidi
*Lophosoria quadripinnata* (Gmel.) C. Chr.	*Terminalia nipensis* Alain
*Lycopodium dichotomum* Jacq.	*Trema cubensis* Urb.
*Lyonia calycosa* (Samll) Urb.	*Trichomanes crispum* L.
*Lyonia maestrensis* Acuña and Roig	*Vaccinium leonis* Acuña & Roig
*Magnolia cubensis* Urb.	*Vandenboschia scandens* (L.) Copel
*Marcgravia evenia* Kr et Urb.	*Vanilla phaeantha* Rchb.
*Mataiba domingensis* (DC.) Radlk.	*Vanilla wrightii* Rchb.
*Mecodium polyanthos* (Sw.) Copel	*Vernonia parvuliceps* Ekm.
*Meriania leucantha* Sw. subsp. *nana* (Naud.) Borhidi	*Viburnum villosum* Sw.
*Meringium fucoides* (Sw.) Copel	*Wallenia laurifolia* (A. Rich.) Wr in Sauv subsp. *pinetorum* (Britt.) Borhidi
*Miconia acunae* Borhidi	*Wigandia reflexa* Brand
*Miconia alternifolia* Griseb.	*Zanthoxyllum cubense* P. Wils.
*Miconia dodecandra* (Desv.) Cogn.	

**Table 2:** Plant species found with our studies in the Dominican Republic (DR) and not present in the relevés from Cuba [31,32].

*Alsophila minor* (D.C.Eaton) R.M.Tryon	*Magnolia hamorii* Howard
*Anacheilium cochleatum* (L.) Hoffm.	*Magnolia pallescens* Urb. & Ekm.
*Antrophyum lanceolatum* (L.) Kaulf.	*Malpighia macracantha* Ekm. & Nied.
*Arthrostylidium multispicatum* Pilger	*Marattia kaulfussii* J. Smith
*Arthrostylidium sarmentosum* Pilger	*Marcgravia rubra* A. Liogier
*Asplenium radicans* L.	*Maxillaria coccinea* (Jacq.) L.O. Wms.
*Baccharis myrsinites* (Lam.) Pers.	*Mecranium ovatum* Cog.
*Bactris plumeriana* Mart.	*Meriania involucrata* (Desv.) Naud.
*Beilschmiedia pendula* (Sw.) Hemsl.	*Miconia mirabilis* (Aubl.) L.O. Willians
*Blechneum fragile* (Liebm.) Morton & Lellinger	*Miconia prasina* (Sw.) DC.
*Blechnum tuerckheimii* A. Brause	*Miconia racemosa* (Aubl.) DC.
*Bocconia frutescens* L.	*Microgramma piloselloides* L.
*Buchenavia tetraphylla* (Aubl.) R.A. Howard	*Mikania cordifolia* (L.) Willd.
*Byrsonima lucida* (Mill.) L.C. Rich.	*Mikania venosa* A. Liogier
*Byrsonima spicata* (Cav.) Kunth	*Mimosa pudica* L.
*Calyptrantes selleanus* Urb. & Ekm.	*Mucuna urens* (L.) Fawc. & Rendle
*Camparettia falcata* Poepp. & Endl.	*Myrcia deflexa* (Poir) DC.
*Casearea arborea* (L.C. Rich.) Urb.	*Myrsine nubicola* A. Liogier
*Cecropia screberiana* Miq.	*Nephrolepis multiflora* (Roxb.) Jarret
*Cestrum coelophlebium* O. E. Schulz	*Nepsera aquatica* (Aubl.) Naud.
*Cestrum daphnoides* Griseb.	*Neurolaena lobata* (L.) Cass.
*Cestrum inclusum* Urb.	*Niphidium crassifolium* (L.) Lell.
*Cinnamomum alainii* (C.K. Allen) A. Liogier	*Ocotea acarina* C. K. Allen
*Cissampelos pareira* L.	*Ocotea floribunda* (Sw.) Mez
*Cissus verticillata* (L.) Nicholson & Farris	*Ocotea foeniculacea* Mez
*Clidemia umbellata* (Miller) L.O. Wms.	*Ocotea globosa* (Aubl.) Schlecht. & Cham.
*Clusia clusioides* (Griseb.) D’Arcy	*Ocotea nemodaphne* Mez
*Cnemidaria horrida* (L.) K. Presl	*Ocotea patens* (Sw.) Nees
*Coccoloba wrightii* Lindau	*Odontadenia polyneura* (Urb.) Wood.
*Columnea domingensis* (Urb.) Wiehler	*Olyra latifolia* L.
*Columnea sanguinea* Urb.	*Oncidium variegatum* (Sw.) Sw.
*Commelina elegans* Kunth	*Ophioglossum palmatum* L.
*Cordia dependens* Urb. & Ekm.	*Oreopanax capitatus* (Jacq.) Decne. & Planch.
*Cupania americana* L.	*Ormosia krugii* Urb.
*Cyathea fulgens* C. Chr.	*Palicourea crocea* (Sw.) Schultes
*Cyathea furfuracea* Baker	*Passiflora rubra* L.
*Chaetocarpus domingensis* Proctor	*Peperomia hernandifolia* (Vahl) A. Dietr.
*Daphnosis crassifolia* (Poir.) Meiss.	*Persea krugii* Mez
*Dendropanax arboreus* (L.) Dcne & Planch.	*Persea oblongifolia* Kopp.
*Dichaea glauca* (Sw.) Lindley	*Phlebodium aureum* (L.) J. Smith
*Didymopanax tremulus* Krug. & Urb.	*Pilea geminata* Urb.
*Dilomilis montana* (Sw.) Summerh.	*Pinguicula casabitoana* J. Jiménez
*Diplazium hastile* (Christ.) C. Chr.	*Piper adunculum* L.
*Diplazium hians* Kuntze	*Pleurothalis ruscifolia* (Jaq.) R. Br.
*Ditta maestrensis* Borhidi	*Pleurothallis domingensis* Cogn.
*Elaphoglossum crinitum* (L.) C. Chr.	*Polygala fuertesii* (Urb.) Blake
*Elaphoglossum latifolium* (Sw.) J. Sm.	*Polypodium angustifolium* Sw.
*Elleanthus cephalotus* Garay & Sweet	*Polypodium loriceum* L.
*Entada gigas* (L.) Fawc. & Rendle	*Pothomorphe peltata* (L.) Miquel
*Epidendrum anceps* Jacq.	*Pothuya nudicaulis* (L.) Regel
*Epidendrum carpophorum* Barb. Rodr.	*Prestoea montana* (Grah.) Nichol
*Epidendrum jamaicense* Lindl	*Psychotria domingensis* Jacq.
*Epidendrum ramosum* Jacq.	*Psychotria liogieri* Sateyerm
*Eupatorium odoratum* L.	*Psychotria uliginosa* Sw.
*Exostema elliptica* Griseb.	*Pytirogramma calomelanos* (L.) Link
*Gleychenia bifida* (Willd.) Spreng.	*Renealmia jamaicensis* (Gaertn.) Horan var. *puberula* (Gagn.) Maas
*Gomedesia lindeniana* Berg.	*Rondeletia ochracea* Urb.
*Gonocalyx tetrapterus* A. Liogier	*Sagraea fuertesii* (Cogn.in Urb.) Alain
*Grammitis asplenifolia* (L.) Proctor	*Schlegelia brachyantha* Griseb.
*Guarea guidonea* Sleumer	*Schradera subsessilis* Steyermark
*Guatteria blainii* (Griseb.) Urb.	*Senecio lucens* (Poir.) Urb.
*Guzmania monostrachya* (Sw.) Rusby	*Sloanea berteriana* Choisy
*Gyrotaenia myriocarpa* Griseb.	*Smilax havanensis* Jacq.
*Hedychium coronarium* Koen.	*Smilax populnea* Kunt var. *horrida* O.E. Schulz
*Hedyosmum domingense* Urb.	*Solanum crotonoides* Lam.
*Hirtella triandra* Sw.	*Solanum jamaicense* Mill.
*Hyeronima domingensis* Urb.	*Solanum torvum* Sw.
*Hyeronima montana* A. Liogier	*Solanum virgatum* Lam.
*Hypolepis hispaniolica* Mason	*Stigmaphyllon emarginatum* (L.) A. Juss.
*Hyptis americana* (Poir.) Briq.	*Styrax ochraceus* Urb.
*Ichnanthus pallens* (Sw.) Munro	*Syngonium podophyllum* Schott
*Ilex tuerckheimii* Loes.	*Tabebuia bullata* A. Gentry
*Inga fagifolia* (L.) Willd. ex Benth.	*Tabebuia vinosa* A. Gentry
*Inga vera* Willd.	*Torralbasia cuneifolia* (C. Wright) Krug. & Urb.
*Ipomoea furcyensis* Urb.	*Triunfetta semitriloba* Jacq.
*Ipomoea tiliacea* (Willd.) Choisy	*Turpinia occidentalis* (Sw.) G. Don
*Isachne rigidifolia* (Poir.) Urb.	*Uncinia hamata* (L.) Urb.
*Lasianthus bahorucanus* Zanoni	*Urena lobata* L.
*Leandra limoides* (Urb.) W. Judd & Skean	*Urera baccifera* (L.) Gaud.
*Lobelia robusta* Graham	*Vaccinium racemosum* (Vahl) Wilbur & Luteyn
*Lobelia rotundifolia* Juss.	*Vernonia buxifolia* (Cass.) Less.
*Lomariopsis sorbifolia* (L.) Fée	*Vitis tiliifolia* H. & B. ex Willd.
*Lycopodium cernuum* L.	*Vittaria lineata* (L.) Smith
*Lycopodium clavatum* L.	*Vriesea sintenisii* (Baker) L.B. Smith & Pitt.
*Lyonia alainii* W. Judd.	*Vriesea tuercheimii* (Mez.) L.B. Smith
*Macrocarpaea domingensis* Urb.	*Zanthoxyllum martinicensis* (DC.) Lam.
*Machaerina cubensis* (Kük.) T. Koyama	

**Table 3:** Synthetic table of the four new associations considered in this study.

Species	As1	As2	As3	As4	P
*Myrsine coriacea* (Sw.) R. Br.	IV	III	V	III	4
*Ocotea leucoxylon* (Sw.) Mez	I	IV	III	III	4
*Prestoea montana* (Grah.) Nichol	I	V	V	V	4
*Psychotria domingensis* Jacq.	IV	IV	V	III	4
*Gleychenia bifida* (Willd.) Spreng.	II	I	-	I	3
*Clidemia umbellata* (Miller) L.O. Wms.	I	I	-	I	3
*Renealmia jamaicensis* (Gaertn.) Horan var. *puberula* (Gagn.) Maas	V	III	V	-	3
*Arthrostylidium multispicatum* Pilger	V	IV	III	-	3
*Rondeletia ochracea* Urb.	V	II	V	-	3
*Didymopanax tremulus* Krug. & Urb.	IV	I	V	-	3
*Psychotria guadalupensis* (DC.) Howard	III	III	V	-	3
*Mikania venosa* A. Liogier	II	IV	V	-	3
*Odontosoria uncinella* (Kunze) Fée	II	II	V	-	3
*Brunellia comocladifolia* H. & B.	II	III	III	-	3
*Lobelia rotundifolia* Juss.	III	I	I	-	3
*Alchornea latifolia* Sw.	-	III	I	V	3
*Miconia mirabilis* (Aubl.) L.O. Willians	-	II	I	V	3
*Mucuna urens* (L.) Fawc. & Rendle	-	II	I	V	3
*Nephrolepis multiflora* (Roxb.) Jarret	-	I	III	I	3
*Ilex macfadyenii* (Walp.) Rehder	V	-	I	-	2
*Chionanthus domingensis* Lam.	V	-	I	-	2
*Macrocarpaea domingensis* Urb.	IV	-	III	-	2
*Polygala fuertesii* (Urb.) Blake	IV	-	III	-	2
*Marcgravia rubra* A. Liogier	IV	-	I	-	2
*Alsophila minor* (D. C. Eaton) R. M. Tryon	V	III	-	-	2
*Palicourea alpina* (Sw.) DC.	V	II	-	-	2
*Blechnum occidentale* L.	III	III	-	-	2
*Cyrilla racemiflora* L.	IV	IV	-	-	2
*Ocotea nemodaphne* Mez	III	-	I	-	2
*Schradera subsessilis* Steyermark	II	-	I	-	2
*Lycopodium clavatum* L.	II	-	-	I	2
*Odontadenia polyneura* (urb.) Wood.	II	I	-	-	2
*Byrsonima lucida* (Mill.) DC.	I	II	-	-	2
*Weinmannia pinnata* L.	I	-	V	-	2
*Epidendrum carpophorum* Barb. Rodr.	I	-	I	-	2
*Epidendrum carpophorum* Barb. Rodr.	I	-	I	-	2
*Pleurothallis domingensis* Cogn.	I	II	-	-	2
*Cestrum coelophlebium* O. E. Schulz	I	II	-	-	2
*Olyra latifolia* L.	I	I	-	-	2
*Cecropia screberiana* Miq.	-	III	-	V	2
*Turpinia occidentalis* (Sw.) G. Don	-	III	-	V	2
*Mikania cordifolia* (L.) Willd.	-	I	-	V	2
*Pothomorphe peltata* (L.) Miquel	-	I	-	III	2
*Ichnanthus pallens* (Sw.) Munro	-	III	-	I	2
*Guzmania monostrachya* (Sw.) Rusby	-	II	-	I	2
*Dendropanax arboreus* (L.) Dcne & Planch.	-	IV	I	-	2
*Dichaea glauca* (Sw.) Lindley	-	III	III	-	2
*Epidendrum ramosum* Jacq.	-	III	I	-	2
*Gomedesia lindeniana* Berg.	-	II	V	-	2
*Myrcia deflexa* (Poir) DC.	-	II	V	-	2
*Peperomia hernandifolia* (Vahl) A. Dietr.	-	II	V	-	2
*Vriesea tuercheimii* (Mez.) L.B. Smith	-	I	V	-	2
*Cyathea fulgens* C. Chr.	-	I	V	-	2
*Magnolia hamorii* Howard	-	I	V	-	2
*Mecranium ovatum* Cog.	-	I	V	-	2
*Lasianthus bahorucanus* Zanoni	-	I	V	-	2
*Nephrolepis biserrata* (Sw.) Schott	-	I	V	-	2
*Columnea domingensis* (Urb.) Wiehler	-	I	V	-	2
*Hedyosmum domingense* Urb.	-	I	III	-	2
*Lomariposis sorbifolia* (L.) Feé	-	I	III	-	2
*Beilschmiedia pendula* (Sw.) Hemsl.	-	I	III	-	2
*Vaccinium racemosum* (Vahl) Wilbur & Luteyn	IV	I	III	-	2
*Ocotea acarina* C.	-	I	I	-	2
*Hypolepis hispaniolica* Mason	-	I	I	-	2
*Schlegelia brachyantha* Griseb.	-	I	I	-	2
*Sagraea fuertesii* (Cogn.in Urb.) Alain	-	II	I	-	2
*Niphidium crassifolium* (L.) Lell.	-	I	I	-	2
*Phlebodium aureum* (L.) J. Smith	-	I	I	-	2
*Polypodium loriceum* L.	-	I	I	-	2
*Epidendrum jamaicense* Lindl	-	I	I	-	2
*Microgramma piloselloides* L.	-	I	-	I	2
*Miconia prasina* (Sw.) DC.	-	-	III	V	2
*Guarea guidonea* Sleumer	-	-	I	V	2
*Tibouchina longifolia* (Vahl) Baill.	-	-	I	III	2
*Smilax domingensis* Willd.	-	-	I	I	2
*Magnolia pallescens* Urb. & Ekm.	V	-	-	-	1
*Styrax ochraceus* Urb.	V	-	-	-	1
*Hyeronima montana* A. Liogier	V	-	-	-	1
*Cyathea furfuracea* Baker	V	-	-	-	1
*Clusia clusioides* (Griseb.) D’Arcy	V	-	-	-	1
*Ditta maestrensis* Borhidi	V	-	-	-	1
*Persea oblongifolia* Kopp.	V	-	-	-	1
*Smilax populnea* Kunt var. *horrida* O.E. Schulz	V	-	-	-	1
*Tabebuia vinosa* A. Gentry	V	-	-	-	1
*Gonocalyx tetrapterus* A. Liogier	V	-	-	-	1
*Cinnamomum alainii* (C.K. Allen) A. Liogier	IV	-	-	-	1
*Vriesea sintenisii* (Baker) L.B. Smith & Pitt.	III	-	-	-	1
*Baccharis myrsinites* (Lam.) Pers.	III	-	-	-	1
*Pinguicula casabitoana* J. Jiménez	III	-	-	-	1
*Chaetocarpus domingensis* Proctor	II	-	-	-	1
*Odontosoria aculeata* (L.) J. Sm.	I	-	-	-	1
*Myrsine nubicola* A. Liogier	I	-	-	-	1
*Persea krugii* Mez	I	-	-	-	1
*Lycopodium cernuum* L.	I	-	-	-	1
*Isachne rigidifolia* (Poir.) Urb.	I	-	-	-	1
*Machaerina cubensis* (Kük.) T. Koyama	I	-	-	-	1
*Vernonia buxifolia* (Cass.) Less.	I	-	-	-	1
*Lyonia alainii* W. Judd.	I	-	-	-	1
*Clidemia hirta* (L.) D. don	I	-	-	-	1
*Bocconia frutescens* L.	I	-	-	-	1
*Dilomilis montana* (Sw.) Summerh.	I	-	-	-	1
*Myrcia splendens* (Sw.) DC.	-	IV	-	-	1
*Cissampelos pareira* L.	-	III	-	-	1
*Uncinia hamata* (L.) Urb.	-	III	-	-	1
*Tabebuia bullata* A. Gentry	-	III	-	-	1
*Blechnum tuerckheimii* A. Brause	-	III	-	-	1
*Senecio lucens* (Poir) Urb.	-	III	-	-	1
*Coccoloba wrightii* Lindau	-	III	-	-	1
*Guatteria blainii* (Griseb.) Urb.	-	II	-	-	1
*Solanum crotonoides* Lam.	-	II	-	-	1
*Vitis tiliifolia* H. & B. ex Willd.	-	I	-	-	1
*Anacheilium cochleatum* (L.) Hoffm.	-	I	-	-	1
*Antrophyum lanceolatum* (L.) Kaulf.	-	I	-	-	1
*Camparettia falcata* Poepp. & Endl.	-	I	-	-	1
*Passiflora rubra* L.	-	I	-	-	1
*Smilax havanensis* Jacq.	-	I	-	-	1
*Stigmaphyllon emarginatum* (L.) A. Juss.	-	I	-	-	1
*Commelina elegans* Kunth	-	I	-	-	1
*Diplazium hastile* (Christ.) C. Chr.	-	I	-	-	1
*Diplazium hians* Kuntze	-	I	-	-	1
*Epidendrum anceps* Jacq.	-	I	-	-	1
*Grammitis asplenifolia* (L.) Proctor	-	I	-	-	1
*Jacquiniella globosa* (Jacq.) Schlechter	-	I	-	-	1
*Oncidium variegatum* (Sw.) Sw.	-	I	-	-	1
*Pothuya nudicaulis* (L.) Regel	-	I	-	-	1
*Vittaria lineata* (L.) Smith	-	I	-	-	1
*Cestrum inclusum* Urb.	-	I	-	-	1
*Cordia dependens* Urb. & Ekm.	-	I	-	-	1
*Daphnosis crassifolia* (Poir.) Meiss.	-	I	-	-	1
*Eupatorium odoratum* L.	-	I	-	-	1
*Gyrotaenia myriocarpa* Griseb.	-	I	-	-	1
*Hyptis americana* (Poir.) Briq.	-	I	-	-	1
*Lasianthus lanceolatus* (Griseb.) Gómez Maza	-	I	-	-	1
*Lobelia robusta* Graham	-	I	-	-	1
*Psychotria liogieri* Sateyerm	-	I	-	-	1
*Solanum virgatum* Lam.	-	I	-	-	1
*Pilea geminata* Urb.	-	I	-	-	1
*Exostema elliptica* Griseb.	-	I	-	-	1
*Malpighia macracantha* Ekm. & Nied.	-	I	-	-	1
*Ocotea floribunda* (Sw.) Mez	-	I	-	-	1
*Ocotea patens* (Sw.) Nees	-	I	-	-	1
*Ipomoea furcyensis* Urb.	-	I	-	-	1
*Columnea sanguinea* Urb.	-	-	V	-	1
*Elaphoglossum crinitum* (L.) C. Chr.	-	-	V	-	1
*Elaphoglossum latifolium* (Sw.) J. Sm.	-	-	V	-	1
*Elleanthus cephalotus* Garay & Sweet	-	-	V	-	1
*Pleurothalis ruscifolia* (Jaq.) R. Br.	-	-	V	-	1
*Hyeronima domingensis* Urb.	-	-	V	-	1
*Calyptrantes selleanus* Urb. & Ekm.	-	-	V	-	1
*Torralbasia cuneifolia* (C. Wright) Krug. & Urb.	IV	-	III	-	1
*Meriania involucrata* (Desv.) Naud.	-	-	III	-	1
*Miconia punctata* (Desr.) D. Don	-	-	III	-	1
*Ophioglossum palmatum* L.	-	-	III	-	1
*Blechneum fragile* (Liebm.) Morton & Lellinger	-	-	III	-	1
*Arthrostylidium sarmentosum* Pilger	-	-	III	-	1
*Ilex tuerckheimii* Loes.	-	-	I	-	1
*Leandra limoides* (Urb.) W. Judd & Skean	-	-	I	-	1
*Maxillaria coccinea* (Jacq.) L.O. Wms.	-	-	I	-	1
*Asplenium radicans* L.	-	-	I	-	1
*Cestrum daphnoides* Griseb.	-	-	I	-	1
*Polypodium angustifolium* Sw.	-	-	I	-	1
*Ocotea foeniculacea* Mez	-	-	I	-	1
*Hillia parasitica* Jacq.	-	-	I	-	1
*Marattia kaulfussii* J. Smith	-	-	I	-	1
*Buchenavia tetraphylla* (Aubl.) R. A. Howard	-	-	-	V	1
*Byrsonima spicata* (Cav.) Kunth	-	-	-	V	1
*Casearea arborea* (L. C. Rich.) Urb.	-	-	-	V	1
*Clusia rosea* Jacq.	-	-	-	V	1
*Cyathea arborea* (L.) J.E. Smith	-	-	-	V	1
*Didymopanax morototoni* (Aubl.) Decne. & Planch	-	-	-	V	1
*Pytirogramma calomelanos* (L.) Link	-	-	-	V	1
*Miconia serrulata* (DC.) Naud.	-	-	-	V	1
*Ocotea globosa* (Aubl.) Schlecht. & Cham.	-	-	-	V	1
*Oreopanax capitatus* (Jacq.) Decne. & Planch.	-	-	-	V	1
*Ormosia krugii* Urb.	-	-	-	V	1
*Sloanea berteriana* Choisy	-	-	-	V	1
*Cnemidaria horrida* (L.) K. Presl	-	-	-	V	1
*Solanum torvum* Sw.	-	-	-	V	1
*Ipomoea tiliacea* (Willd.) Choisy	-	-	-	V	1
*Inga fagifolia* (L.) Willd. ex Benth.	-	-	-	III	1
*Inga vera* Willd.	-	-	-	III	1
*Bactris plumeriana* Mart.	-	-	-	III	1
*Nepsera aquatica* (Aubl.) Naud.	-	-	-	III	1
*Syngonium podophyllum* Schott	-	-	-	III	1
*Psychotria uliginosa* Sw.	-	-	-	III	1
*Urera baccifera* (L.) Gaud.	-	-	-	III	1
*Mimosa pudica* L.	-	-	-	I	1
*Neurolaena lobata* (L.) Cass.	-	-	-	I	1
*Triunfetta semitriloba* Jacq.	-	-	-	I	1
*Cupania americana* L.	-	-	-	I	1
*Hirtella triandra* Sw.	-	-	-	I	1
*Miconia racemosa* (Aubl.) DC.	-	-	-	I	1
*Zantoxylum martinicensis* (Lam.) DC.	-	-	-	I	1
*Cissus verticillata* (L.) Nicholson & Farris	-	-	-	I	1
*Entada gigas* (L.) Fawc. & Rendle	-	-	-	I	1
*Palicourea crocea* (Sw.) Schultes	-	-	-	I	1
*Piper adunculum* L.	-	-	-	I	1
*Coccocypselum herbaceum* Aubl.	-	-	-	I	1
*Hedychium coronarium* Koen.	-	-	-	I	1
*Solanum jamaicense* Mill.	-	-	-	I	1
*Urena lobata* L.	-	-	-	I	1

As1: *Hyeronimo montanae-Magnolietum pallescenti*. As2: *Cyatheo furfuracei-Prestoetum montanae*. As3: *Hyeronimo dominguensis-Magnolietum hamorii*. As4: *Ormosio krugii-Prestoetum montanae. P: presences*.

**Table 4:** Shannon diversity by 17 relevé from Dominican Republic (DR).

	DR1	DR2	DR3	DR4	DR5	DR6	DR7	DR8	DR9	DR10	DR11	DR12	DR13	DR14	DR15	DR16	DR17
Shannon_T	3.612	3.443	2.451	3.566	3.464	3.557	3.557	3.458	3.424	3.389	3.786	3.972	3.781	3.702	3.917	3.496	3.499
Shannon_Ca	3.170	3.247	2.165	3.097	3.119	3.152	2.819	2.606	2.803	2.563	3.119	3.154	3.013	2.683	3.173	3.061	3.027
Shannon_Co	2.591	1.718	1.099	2602	2.232	2.507	2.910	2901	2.655	2.814	3.066	3.391	3.162	3.256	3.277	2.458	2.532
Shannon_Ne	3.178	2.947	2.160	3.000	2.997	3.119	3.347	3.244	3.104	2.998	3.561	3.601	3.388	3.458	3.897	3.435	3.438
Shannon_E	2.574	2.508	1.089	2.732	2.490	2.557	1.891	1.842	2.137	2.158	2.410	2.803	2.658	2.074	0.000	0.000	0.000

Shannon_T = total diversity; Shannon_Ca = characteristic community species diversity; Shannon_Co = companion community species diversity; Shannon_Ne = non-endemic species diversity; Shannon_E = endemic species diversity.

**Table 5:** Diversity analysis of each of the four plant associations.

	As1	As2	As3	As4
**Shannon_T**	3.049	2.681	3.268	2.400
**Shannon_Ca**	2.743	2.533	3.105	2.921
**Shannon_Co**	2.330	2.475	3.218	2.755
**Shannon_Ne**	2.810	2.397	2.994	1.795
**Shannon_E**	2.572	1.823	2.486	0.000

As1:* Hyeronimo montanae-Magnolietum pallescentis*. As2: *Cyatheo furfuracei-Prestoetum motanae*. As3: *Hyeronimo dominguensis-Magnolietum hamorii*. As4: *Ormosio krugii-Prestoetum montanae*. Shannon_T = total diversity; Shannon_Ca = characteristic community species diversity; Shannon_Co = companion community species diversity; Shannon_Ne = non-endemic species diversity; Shannon_E = endemic species diversity.

**Table 6:** Plant communities studied and number of relevés for each.

	Plant Communities	Authors	N. of Relevés
C1	*Clethro*-*Pinetum maestrensis* Borhidi 1991 (Cuba)	Borhidi [31]. Table 139, page 624	5
C2	*Hyeronimo*-*Sloanetum curatellifoliae* Borhidi 1991 (Cuba)	Borhidi [31]. Table 140, page 627	5
C3	*Alchorneo*-*Calophylletum rivularis* Reyes 2005 (Cuba)	Reyes [32]. Table 1	6
C4	*Pruno*-*Guareetum guidoniae* Reyes & Acosta 2011 (Cuba)	Reyes & Acosta [35]. Table 2	4
C5	*Ocoteo*-*Phoebietum elongatae* Reyes & Acosta 2010 (Cuba)	Reyes & Acosta [33]. Table 1	7
C6	*Guareo guidoniae*-*Zantoxyletum martinicensis* Reyes & Acosta 2010 (Cuba)	Reyes & Acosta [34]. Table 1	8
			Total 35 relevés
DR7	*Hyeronimo montanae*-*Magnolietum pallescentis nova* (DR)	Own relevés	5
DR8	*Cyatheo furfuracei*-*Prestoetum montanae nova* (DR)	Own relevés	5
DR9	*Hyeronimo dominguensis*-*Magnolietum hamorii nova* (DR)	Own relevés	4
DR10	*Ormosio krugii*-*Prestoetum montanae nova* (DR)	Own relevés	3
DR11	Vegetation relevés (DR)	May & Peguero [10] Table 1 page 23	3
DR12	Vegetation relevés (DR)	May [9] Table 1 page 171	12
			Total 32 relevés

C1–C6 Cuba, DR7–DR12 Dominican Republic.

## Appendix A

**Table A1:** Association Hyeronimo montanae-Magnolietum pallescentis.

N° rel.				4	5	10	11	12
**N° order**				DR1	DR2	DR4	DR5	DR6
**Altitude**				1481	1474	1473	1441	1465
**Area in m^2^** **× 10**				200	100	200	50	200
**Cover ratio In %**				100	90	100	100	100
**Xn in m.**				15	15	10	4	20
**Characteristics of the association and higher units**	**Family**	**Biotype**	**Status**					
*Magnolia pallescens* Urb. & Ekm.	Magnoliaceae	A	E	3	3	5	1	4
*Cyathea furfuracea* Baker	Cyatheaceae	A	N	2	3	2	4	2
*Chionanthus domingensis* Lam.	Oleaceae	A	N	2	3	3	1	2
*Gonocalyx tetrapterus* A. Liogier	Ericaceae	Tr	E	1	2	3	1	2
*Hyeronima montana* A. Liogier	Euphorbiaceae	A	E	+	3	2	4	4
*Didymopanax tremulus* Krug. & Urb.	Araliaceae	A	E	5	2	3	-	5
*Persea oblongifolia* Kopp.	Lauraceae	A	E	2	2	3	1	3
*Arthrostylidium multispicatum* Pilger	Poaceae	Tr	E	2	3	2	1	2
*Rondeletia ochracea* Urb.	Rubiaceae	A	E	1	1	2	3	3
*Alsophila minor* (D.C. Eaton) R.M. Tryon	Cyatheaceae	A	N	2	2	2	2	2
*Tabebuia vinosa* A. Gentry	Bignoniaceae	A	E	1	+	1	1	+
*Ditta maestrensis* Borhidi	Euphorbiaceae	A	N	1	2	3	2	2
*Smilax populnea* Kunt var. *horrida* O.E. Schulz	Smilacaceae	Tr	N	1	3	1	1	+
*Ilex macfadyenii* (Walp.) Rehder	Aquifoliaceae	A	N	1	3	+	+	+
*Clusia clusioides* (Griseb.) D’Arcy	Clusiaceae	A	N	+	1	1	1	2
*Cyrilla racemiflora* L.	Cyrillaceae	A	N	2	2	3	-	2
*Vaccinium racemosum* (Vahl) Wilbur & Luteyn	Ericaceae	Tr	N	2	3	-	1	1
*Cinnamomum alainii* (C.K. Allen) A. Liogier	Lauraceae	A	E	-	+	2	1	2
*Marcgravia rubra* A. Liogier	Marcgraviaceae	Tr	E	1	1	2	-	2
*Myrsine coriacea* (Sw.) R. Br.	Myrsinaceae	A	N	1	2	+	+	-
*Pinguicula casabitoana* J. Jiménez	Lentibulariaceae	Ep	E	+	+	1	-	-
*Vriesea sintenisii* (Baker) L.B. Smith & Pitt.	Bromeliaceae	Ep	N	-	-	2	1	2
*Ocotea nemodaphne* Mez	Lauraceae	A	N	+	1	-	-	2
*Brunellia comocladifolia* H. & B.	Brunelliaceae	A	N	1	+	-	-	-
*Ocotea leucoxylon* (Sw.) Mez	Lauraceae	A	N	1	-	-	-	+
*Schradera subsessilis* Steyermark	Rubiaceae	Tr	N	1	-	2	-	-
*Mikania venosa A. Liogier*	Asteraceae	Tr	E	-	2	-	-	+
*Chaetocarpus domingensis* Proctor	Euphorbiaceae	A	E	-	-	1	-	+
*Odontadenia polyneura* (urb.) Wood.	Apocynaceae	Tr	E	-	-	-	+	+
*Myrsine nubicola* A. Liogier	Myrsinaceae	A	E	+	-	-	-	-
*Prestoea montana* (Grah.) Nichol	Arecaceae	A	N	-	2	-	-	-
*Weinmannia pinnata* L.	Cunoniaceae	A	N	-	+	-	-	-
*Odontosoria uncinella* (Kunze) Fée	Polypodiaceae	Tr	N	-	+	-	-	-
*Persea krugii* Mez	Lauraceae	A	N	-	-	-	1	-
*Epidendrum carpophorum* Barb. Rodr.	Orchidaceae	Ep	N	-	-	-	+	-
*Pleurothallis domingensis* Cogn.	Orchidaceae	Ep	E	-	-	-	+	-
*Byrsonima lucida* (Mill.) L.c. rich.	Malpighiaceae	A	N	-	-	-	+	-
*Dilomilis montana* (Sw.) Summerh.	Orchidaceae	Ep	N	-	-	-	+	-
**Companions species**
*Styrax ochraceus* Urb.	Styracaceae	Ar	E	1	1	1	1	1
*Palicourea alpina* (Sw.) DC.	Rubiaceae	Ar	N	1	3	1	1	+
*Torralbasia cuneifolia* (C. Wright) Krug. & Urb.	Celastraceae	Ar	N	-	+	4	2	3
*Macrocarpaea domingensis* Urb.	Gentianaceae	Ar	E	1	-	+	1	2
*Psychotria domingensis* Jacq.	Rubiaceae	Ar	N	3	-	1	1	+
*Polygala fuertesii* (Urb.) Blake	Polygalaceae	Ar	E	1	-	2	5	-
*Psychotria guadalupensis* (DC.) Howard	Rubiaceae	Ar	N	+	3	-	-	+
*Baccharis myrsinites* (Lam.) Pers.	Asteraceae	Ar	N	-	-	1	1	+
*Bocconia frutescens* L.	Papaveraceae	Ar	N	+	-	-	-	-
*Clidemia umbellata* (Miller) L.O. Wms.	Melastomataceae	Ar	N	+	-	-	-	-
*Vernonia buxifolia* (Cass.) Less.	Asteraceae	Ar	N	+	-	-	-	-
*Cestrum coelophlebium* O. E. Schulz	Solanaceae	Ar	E	-	+	-	-	-
*Lyonia alainii* W. Judd.	Ericaceae	Ar	E	-	-	1	-	-
*Clidemia hirta* (L.) D. don	Melastomataceae	Ar	N	-	-	+	-	-
*Renealmia jamaicensis* (Gaertn.) Horan var. *puberula* (Gagn.) Maas	Zingiberaceae	H	N	+	3	1	1	+
*Lobelia rotundifolia* Juss.	Campanulaceae	H	E	1	-	1	-	+
*Gleychenia bifida* (Willd.) Spreng.	Gleycheniaceae	H	N	2	-	1	-	
*Blechnum occidentale* L.	Blechnaceae	H	N	+	-	1	-	+
*Lycopodium clavatum* L.	Lycopodiaceae	H	N	-	-	1	-	+
*Peperomia hernandifolia* (Vahl) A. Dietr.	Piperaceae	H	N	-	-	-	+	-
*Lycopodium cernuum* L.	Lycopodiaceae	H	N	2	-	-	-	-
*Odontosoria aculeata* (L.) J. Sm.	Polypodiaceae	H	N	-	-	-	+	-
*Isachne rigidifolia* (Poir.) Urb.	Poaceae	H	N	-	-	-	-	1
*Machaerina cubensis* (Kük.) T. Koyama	Cyperaceae	H	N	-	-	-	-	+

Sites sampled. DR1—Casabito. Ébano Verde (19340280E/2105321N). DR2—Casabito (19340299E/2105967N). DR4—Casabito. Ébano Verde (19340283N/2106095N). DR5—Casabito. Ébano Verde (19340288E/2106283N). DR6—Palmerito. Ébano Verde (19340165E/2106429N). Tree = A; Shrub = Ar; Climber = Tr; Epiphyte = Ep; Herb = H; Native = N; Endemic = E; Xn = Average height of the dominant species.

**Table A2:** Association Cyatheo furfuracei-Prestoetum motanae.

N° rel.					6	13	14	15	17
**N° order**					DR3	DR7	DR8	DR9	DR10
**Altitude**					1097	1373	1377	1251	1200
**Area in m^2^** **× 10**					200	50	100	100	50
**Cover ratio In %**					100	100	100	100	100
**Xn in m.**					20	9	9	15	7
**Characteristics of the association and higher units**	**Family**	**Biotype**		**Status**					
*Prestoea montana* (Grah.) Nichol	Arecaceae		A	N	5	4	5	5	4
*Arthrostylidium multispicatum* Pilger	Poaceae		Tr	E	2	3	2	1	2
*Cyathea furfuracea* Baker	Cyatheaceae		A	N	2	1	2	2	+
*Dendropanax arboreus* (L.) Dcne & Planch.	Araliaceae		A	N	2	-	+	+	+
*3Alsophila minor* (D.C. Eaton) R.M. Tryon	Cyatheaceae		A	N	1	1	2	1	-
*Ocotea leucoxylon* (Sw.) Mez	Lauraceae		A	N	-	+	+	+	+
*Coccoloba wrightii* Lindau	Polygnonaceae		A	N	-	1	+	2	+
*Alchornea latifolia* Sw.	Euphorbiaceae		A	N	2	+	-	1	-
*Turpinia occidentalis* (Sw.) G. Don	Staphyleaceae		A	N	-	-	+	2	1
*Brunellia comocladifolia* H. & B.	Brunelliaceae		A	N	2	-	-	-	+
*Byrsonima lucida* (Mill.) L.c. Rich.	Malpighiaceae		A	N	-	1	-	-	+
*Calyptrantes selleanus* Urb. & Ekm.	Myrtaceae		A	E	-	-	-	-	+
*Cecropia screberiana* Miq.	Moraceae		A	N	2	-	2	-	-
*Dichaea glauca* (Sw.) Lindley	Orchidaceae		Ep	N	-	+	-	+	1
*Epidendrum anceps* Jacq.	Orchidaceae		Ep	N	-	1	-	-	-
*Epidendrum jamaicense* Lindl	Orchidaceae		Ep	N	-	-	-	+	-
*Epidendrum ramosum* Jacq.	Orchidaceae		Ep	N	-	-	+	+	-
*Epidendrum ramosum* Jacq.	Orchidaceae		Ep	N	-	-	-	-	-
*Grammitis asplenifolia* (L.) Proctor	Grammitidaceae		Ep	N	-	+	-	-	-
*Guatteria blainii* (Griseb.) Urb.	Annonaceae		A	N	-	+	-	-	+
*Guzmania monostrachya* (Sw.) Rusby	Bromeliaceae		Ep	N	-	+	+	-	-
*Malpighia macracantha* Ekm. & Nied.	Malpighiaceae		A	E	-	-	-	2	-
*Jacquiniella globosa* (Jacq.) Schlechter	Orchidaceae		Ep	N	-	+	-	-	-
*Didymopanax tremulus* Krug. & Urb.	Araliaceae		A	E	1	-	-	-	-
*Miconia mirabilis* (Aubl.) L.O. Willians	Melastomataceae		A	N	-	+	-	-	-
*Exostema elliptica* Griseb.	Rubiaceae		A	N	-	-	+	-	-
*Microgramma piloselloides* L.	Polypodiaceae		Ep	N	-	-	+	-	-
*Camparettia falcata* Poepp. & Endl.	Orchidaceae		Ep	N	-	-	-	+	-
*Antrophyum lanceolatum* (L.) Kaulf.	Adiantaceae		Ep	N	-	-	+	-	-
*Myrsine coriacea* (Sw.) R. Br.	Myrsinaceae		A	N	-	+	-	-	+
*Niphidium crassifolium* (L.) Lell.	Polypodiaceae		Ep	N	-	-	+	-	-
*Oncidium variegatum* (Sw.) Sw.	Orchidaceae		Ep	N	-	-	-	+	-
*Ophioglossum palmatum* L.	Ophioglossaceae		Ep	N	-	-	-	-	-
*Phlebodium aureum* (L.) J. Smith	Polypodiaceae		Ep	N	-	-	-	+	-
*Pleurothallis domingensis* Cogn.	Orchidaceae		Ep	E	-	+	-	-	+
*Pothuya nudicaulis* (L.) Regel	Bromeliaceae		Ep	N	-	-	-	+	-
*Rondeletia ochracea* Urb.	Rubiaceae		A	E	-	+	-	3	-
**Companions species**									
*Myrcia splendens* (Sw.) DC.	Myrtaceae		Ar	N	-	5	2	2	5
*Psychotria domingensis* Jacq.	Rubiaceae		Ar	N	-	3	3	1	1
*Tabebuia bullata* A. Gentry	Bignoniaceae		Ar	E	1	-	+	+	+
*Blechnum tuerckheimii* A. Brause	Blechnaceae		H	E	-	1	2	3	-
*Psychotria guadalupensis* (DC.) Howard	Rubiaceae		Ar	N	-	3	-	1	1
*Renealmia jamaicensis* (Gaertn.) Horan var. *puberula* (Gagn.) Maas	Zingiberaceae		H	N	-	2	2	-	+
*Mikania venosa* A. Liogier	Asteraceae		Tr	E	-	-	+	+	2
*Sagraea fuertesii* (Cogn.in Urb.) Alain	Melastomataceae		Ar	E	-	1	-	-	1
*Senecio lucens* (Poir) Urb.	Asteraceae		Tr	E	-	-	+	2	1
*Smilax havanensis* Jacq.	Smilacaceae		Tr	N	-	+	-	-	-
*Solanum crotonoides* Lam.	Solanaceae		Ar	N	-	1	-	-	+
*Solanum virgatum* Lam.	Solanaceae		Ar	N	-	-	+	-	-
*Stigmaphyllon emarginatum* (L.) A. Juss.	Malpighiaceae		Tr	N	-	-	-	-	+
*Uncinia hamata* (L.) Urb.	Cyperaceae		H	N	-	+	+	+	-
*Vaccinium racemosum* (Vahl) Wilbur & Luteyn	Ericaceae		Tr	N	-	+	-	-	-
*Vitis tiliifolia* H. & B. ex Willd.	Vitaceae		Tr	N	-	-	-	-	+
*Vittaria lineata* (L.) Smith	Pteridaceae		Ep	N	-	-	+	-	-
*Blechnum occidentale* L.	Blechnaceae		H	N	-	1	2	-	+
*Cestrum coelophlebium* O. E. Schulz	Solanaceae		Ar	E	-	-	-	1	+
*Cestrum inclusum* Urb.	Solanaceae		Ar	E	-	-	5	-	-
*Cissampelos pareira* L.	Menispermiaceae		Tr	N	1	-	+	-	-
*Commelina elegans* Kunth	Commelinaceae		H	N	-	-	+	-	-
*Daphnosis crassifolia* (Poir.) Meiss.	Thymelaeaceae		Ar	N	-	+	-	-	-
*Diplazium hastile* (Christ.) C. Chr.	Athyriaceae		H	N	-	-	2	-	-
*Diplazium hians* Kuntze	Athyriaceae		H	N	-	-	-	2	-
*Gleychenia bifida* (Willd.) Spreng.	Gleycheniaceae		H	N	1	-	-	-	+
*Gomedesia lindeniana* Berg.	Myrtaceae		Ar	N	-	-	-	-	1
*Gyrotaenia myriocarpa* Griseb.	Urticaceae		Ar	N	-	-	+	-	-
*Hyptis americana* (Poir.) Briq.	Lamiaceae		Ar	N	-	+	-	-	-
*Ichnanthus pallens* (Sw.) Munro	Poaceae		H	N	-	1	+	+	-
*Ipomoea furcyensis* Urb.	Convolvulaceae		Tr	E	-	-	-	+	-
*Lasianthus lanceolatus* (Griseb.) Gómez Maza	Rubiaceae		Ar	N	-	1	-	-	-
*Lobelia robusta* Graham	Campanulaceae		Ar	E	-	-	+	-	-
*Lobelia rotundifolia* Juss.	Campanulaceae		H	E	-	+	-	-	-
*Odontadenia polyneura* (urb.) Wood.	Apocynaceae		Tr	E	-	-	-	-	1
*Odontosoria uncinella* (Kunze) Fée	Polypodiaceae		Tr	N	-	+	-	-	-
*Olyra latifolia* L.	Poaceae		H	N	-	-	-	+	-
*Palicourea alpina* (Sw.) DC.	Rubiaceae		Ar	N	-	+	-	-	+
*Peperomia hernandifolia* (Vahl) A. Dietr.	Piperaceae		H	N	-	+	-	-	-
*Pilea geminata* Urb.	Urticaceae		H	E	-	-	2	-	-
*Polypodium loriceum* L.	Polypodiaceae		Ep	N	-	-	-	+	-
*Pothomorphe peltata* (L.) Miquel	Piperaceae		Ar	N	-	-	+	-	-
*Mucuna urens* (L.) Fawc. & Rendle	Fabaceae		Tr	N	-	+	-	-	-
*Myrcia deflexa* (Poir) DC.	Myrtaceae		Ar	N	-	-	-	1	+

Sites sampled. DR3—Río Jatubei (19341984E/2105891N). DR7—Camino Casabito al Arroyazo (10339971E/2105962N). DR8—Bajada Casabito al Centro Fernándo Dominguez (19339590E/2105699N). DR9—Casabito-Arroyazo (Ébano Verde) (19339203E/2105784N). DR10—Near Arroyazo (19339203E/2105785N). Tree = A; Shrub = Ar; Climber = Tr; Epiphyte = Ep; Herb = H; Native = N; Endemic = E; Xn = Average height of the dominant species.

**Table A3:** Association Hyeronimo dominguensis-Magnolietum hamorii.

N° rel.				23	24	25	26
**N° order**				DR11	DR12	DR13	DR14
**Altitude**				1207	1239	1233	1140
**Area in m^2^** **× 10**				200	200	200	200
**Cover ratio In %**				100	100	100	100
**Xn i** **n m.**				25	15	20	15
**Characteristics of the association and higher units**	**Family**	**Biotype**	**Status**				
*Magnolia hamorii* Howard	Magnoliaceae	A	E	5	2	2	5
*Hyeronima domingensis* Urb.	Euphorbiaceae	A	E	5	2	5	+
*Cyathea fulgens* C. Chr.	Cyatheaceae	A	N	2	2	2	1
*Myrsine coriacea* (Sw.) R. Br.	Myrsinaceae	A	N	1	1	1	2
*Didymopanax tremulus* Krug. & Urb.	Araliaceae	A	E	+	5	2	3
*Brunellia comocladifolia* H. & B.	Brunelliaceae	A	N	2	1	-	-
*Prestoea montana* (Grah.) Nichol	Arecaceae	A	N	+	2	2	3
*Beilschmiedia pendula* (Sw.) Hemsl.	Lauraceae	A	N	2	-	1	-
*Ocotea leucoxylon* (Sw.) Mez	Lauraceae	A	N	-	1	1	+
*Calyptrantes selleanus* Urb. & Ekm.	Myrtaceae	A	E	+	1	1	-
*Weinmannia pinnata* L.	Cunoniaceae	A	N	2	2	2	-
*Pleurothalis ruscifolia* (Jaq.) R. Br.	Orchidaceae	Ep	N	1	2	2	-
*Elleanthus cephalotus* Garay & Sweet	Orchidaceae	Ep	N	2	2	1	-
*Elaphoglossum crinitum* (L.) C. Chr.	Lomariopsidaceae	Ep	N	1	1	+	-
*Columnea sanguinea* Urb.	Gesneriaceae	ArEp	N	1	2	1	-
*Elaphoglossum latifolium* (Sw.) J. Sm.	Lomariopsidaceae	Ep	N	2	2	2	-
*Miconia prasina* (Sw.) DC.	Melastomataceae	A	N	1	-	1	-
*Rondeletia ochracea* Urb.	Rubiaceae	A	E	1	1	1	-
*Alchornea latifolia* Sw.	Euphorbiaceae	A	N	1	-	-	+
*Dendropanax arboreus* (L.) Dcne & Planch.	Araliaceae	A	N	1	-	-	-
*Miconia mirabilis* (Aubl.) L.O. Willians	Melastomataceae	A	N	-	1	-	+
*Epidendrum ramosum* Jacq.	Orchidaceae	Ep	N	-	-	2	+
*Ophioglossum palmatum* L.	Ophioglossaceae	Ep	N	+	1	-	-
*Ocotea acarina* C.K. Allen	Lauraceae	A	E	-	-	2	1
*Chionanthus domingensis* Lam.	Oleaceae	A	N	-	-	2	-
*Ocotea nemodaphne* Mez	Lauraceae	A	N	-	1	-	-
*Ilex macfadyenii* (Walp.) Rehder	Aquifoliaceae	A	N	-	1	-	-
*Niphidium crassifolium* (L.) Lell.	Polypodiaceae	Ep	N	2	-	-	-
*Polypodium loriceum* L.	Polypodiaceae	Ep	N	1	-	-	-
*Epidendrum jamaicense* Lindl	Orchidaceae	Ep	N	-	2	-	-
*Phlebodium aureum* (L.) J. Smith	Polypodiaceae	Ep	N	1	-	-	-
*Dichaea glauca* (Sw.) Lindley	Orchidaceae	Ep	N	-	2	-	-
*Epidendrum carpophorum* Barb. Rodr.	Orchidaceae	Ep.	N	-	-	1	-
*Ocotea floribunda* (Sw.) Mez	Lauraceae	A	N	-	-	-	1
*Anacheilium cochleatum* (L.) Hoffm.	Orchidaceae	Ep	N	-	-	-	+
*Ocotea patens* (Sw.) Nees	Lauraceae	A	N	-	-	-	+
*Guarea guidonea* Sleumer	Meliaceae	A	N	1	-	-	-
*Maxillaria coccinea* (Jacq.) L.O. Wms.	Orchidaceae	Ep	N	-	2	-	-
*Ocotea foeniculacea* Mez	Lauraceae	A	N	-	1	-	-
*Cecropia screberiana* Miq.	Moraceae	A	N	-	-	-	1
*Beilschmiedia pendula* (Sw.) Hemsl.	Lauraceae	A	N	-	-	-	1
**Companions species**							
*Psychotria domingensis* Jacq.	Rubiaceae	Ar	N	2	2	2	1
*Mikania venosa A. Liogier*	Asteraceae	Tr	E	1	2	1	2
*Gomedesia lindeniana* Berg.	Myrtaceae	Ar	N	1	1	1	2
*Lasianthus bahorucanus* Zanoni	Rubiaceae	H	E	2	2	1	1
*Columnea domingensis* (Urb.) Wiehler	Gesneriaceae	Ar	E	2	1	+	1
*Odontosoria uncinella* (Kunze) Fée	Polypodiaceae	Tr	N	3	2	2	2
*Mecranium ovatum* Cog.	Melastomataceae	Ar	E	2	1	1	1
*Vriesea tuercheimii* (Mez.) L.B. Smith	Bromeliaceae	H	E	2	2	2	1
*Nephrolepis biserrata* (Sw.) Schott	Lomariopsidaceae	H	N	2	2	2	2
*Peperomia hernandifolia* (Vahl) A. Dietr.	Piperaceae	H	N	+	1	1	1
*Psychotria guadalupensis* (DC.) Howard	Rubiaceae	Ar	N	2	2	1	-
*Myrcia deflexa* (Poir) DC.	Myrtaceae	Ar	N	2	1	1	2
*Lomariposis sorbifolia* (L.) Feé	Lomariopsidaceae	H	N	1	-	1	1
*Hedyosmum domingense* Urb.	Chloranthaceae	Ar	E	-	1	1	+
*Lomariposis sorbifolia* (L.) Feé	Lomariopsidaceae	H	N	2	2	-	1
*Renealmia jamaicensis* (Gaertn.) Horan var. *puberula* (Gagn.) Maas	Zingiberaceae	H	N	2	1	2	-
*Vaccinium racemosum* (Vahl) Wilbur & Luteyn	Ericaceae	Tr	N	-	1	1	-
*Macrocarpaea domingensis* Urb.	Gentianaceae	Ar	E	-	2	1	-
*Polygala fuertesii* (Urb.) Blake	Polygalaceae	Ar	E	-	1	1	-
*Arthrostylidium multispicatum* Pilger	Poaceae	Tr	E	3	2	-	-
*Torralbasia cuneifolia* (C. Wright) Krug. & Urb.	Celastraceae	Ar	N	-	1	1	
*Mucuna urens* (L.) Fawc. & Rendle	Fabaceae	Tr	N	1	-	-	2
*Schlegelia brachyantha* Griseb.	Schlegeliaceae	Tr	N	1	-	-	+
*Meriania involucrata* (Desv.) Naud.	Melastomataceae	Ar	E	-	1	1	-
*Hypolepis hispaniolica* Mason	Polypodiaceae	Tr	E	-	2	-	1
*Arthrostylidium sarmentosum* Pilger	Poaceae	Tr	N	-	2	2	-
*Blechneum fragile* (Liebm.) Morton & Lellinger	Blechnaceae	H	N	-	2	2	-
*Ilex tuerckheimii* Loes.	Aquifoliaceae	Ar	E	-	-	+	-
*Cordia dependens* Urb. & Ekm.	Boraginaceae	Ar	E	-	-	-	+
*Passiflora rubra* L.	Passifloraceae	Tr	N	-	-	-	+
*Eupatorium odoratum* L.	Asteraceae	Ar	N	-	-	-	+
*Mikania cordifolia* (L.) Willd.	Asteraceae	Tr	N	-	-	-	1
*Psychotria liogieri S*ateyerm	Rubiaceae	Ar	N	-	-	-	+
*Marattia kaulfussii* J. Smith	Marattiaceae	H	N	1	-	-	-
*Asplenium radicans* L.	Aspleniaceae	H	N	1	-	-	-
*Smilax domingensis* Willd.	Smilacaceae	Tr	N	+	-	-	-
*Leandra limoides* (Urb.) W. Judd & Skean	Melastomataceae	Ar	E	-	1	-	-
*Hillia parasitica* Jacq.	Rubiaceae	Tr	N	-	2	-	-
*Cestrum daphnoides* Griseb.	Solanaceae	Ar	E	-	1	-	-
*Tibouchina longifolia* (Vahl) Baill.	Melastomataceae	Ar	N	-	1	-	-
*Clidemia umbellata* (Miller) L.O. Wms.	Melastomataceae	Ar	N	-	-	-	+
*Schradera subsessilis* Steyermark	Rubiaceae	Tr	E	1	-	-	-
*Marcgravia rubra* A. Liogier	Marcgraviaceae	Tr	E	-	-	1	-
*Lobelia rotundifolia* Juss.	Campanulaceae	H	E	-	1	-	-
*Blechnum occidentale* L.	Blechnaceae	H	N	-	-	-	+
*Cissampelos pareira* L.	Menispermiaceae	Tr	N	-	-	-	+
*Myrcia splendens* (Sw.) DC.	Myrtaceae	Ar	N	-	-	-	3
*Ichnanthus pallens* (Sw.) Munro	Poaceae	H	N	-	-	-	1
*Sagraea fuertesii* (Cogn.in Urb.) Alain	Melastomataceae	Ar	E	-	1	-	-

Sites sampled. DR11—Sierra Bahoruco. El Cachote (19267592E/2002124N). DR12—Sierra Bahoruco. El Cachote (19268161E/2002764N). DR13—Sierra Bahoruco. Prox. el Cachote (19268152E/2002964N). DR14—Km. 3 del poblado Cachote (19268736E/2000217N). Tree = A; Shrub = Ar; Climber = Tr; Epiphyte = Ep; Herb = H; Native = N; Endemic = E; Xn = Average height of the dominant species.

**Table A4:** Association Ormosio krugii-Prestoetum montanae.

N° rel.				13	15a	15b
**N° order**				DR15	DR16	DR17
**Altitude**				519	541	530
**Area in m^2^** **× 10**				200	200	200
**Cover ratio In %**				75	100	100
**Xn in** **m.**				15	12	15
**Characteristics of the association and higher units**	**Family**	**Biotype**	**Status**			
*Prestoea montana* (Grah.) Nichol	Arecaceae	A	N	3	4	4
*Cecropia screberiana* Miq.	Moraceae	A	N	3	2	3
*Alchornea latifolia* Sw.	Euphorbiaceae	A	N	2	5	4
*Miconia mirabilis* (Aubl.) L.O. Willians	Melastomataceae	A	N	3	2	2
*Miconia prasina* (Sw.) DC.	Melastomataceae	A	N	1	1	1
*Guarea guidonea* Sleumer	Meliaceae	A	N	+	4	4
*Cyathea arborea* (L.) J.E. Smith	Cyatheaceae	A	N	3	4	4
*Turpinia occidentalis* (Sw.) G. Don	Staphyleaceae	A	N	1	+	1
*Clusia rosea* Jacq.	Clusiaceae	A	N	1	+	+
*Ocotea globosa* (Aubl.) Schlecht. & Cham.	Lauraceae	A	N	2	1	1
*Casearea arborea* (L.C. Rich.) Urb.	Flacourtiaceae	A	N	1	1	+
*Oreopanax capitatus* (Jacq.) Decne. & Planch.	Araliaceae	A	N	2	3	3
*Didymopanax morototoni* (Aubl.) Decne. & Planch	Araliaceae	A	N	2	3	3
*Byrsonima spicata* (Cav.) Kunth	Malpighiaceae	A	N	+	1	1
*Buchenavia tetraphylla* (Aubl.) R. A. Howard	Combretaceae	A	N	1	1	1
*Sloanea berteriana* Choisy	Elaeocarpaceae	A	N	1	1	2
*Ormosia krugii* Urb.	Fabaceae	A	N	2	2	2
*Miconia serrulata* (DC.) Naud.	Melastomataceae	A	N	+	+	1
*Bactris plumeriana* Mart.	Arecaceae	A	E	-	1	1
*Myrsine coriacea* (Sw.) R. Br.	Myrsinaceae	A	N	1	1	-
*Ocotea leucoxylon* (Sw.) Mez	Lauraceae	A	N	-	2	2
*Inga fagifolia* (L.) Willd. ex Benth.	Mimosaceae	A	N	-	+	+
*Inga vera* Willd.	Mimosaceae	A	N	-	+	+
*Cupania americana* L.	Sapindaceae	A	N	2	-	-
*Hirtella triandra* Sw.	Chrysobalanaceae	A	N	+	-	-
*Miconia racemosa* (Aubl.) DC.	Melastomataceae	A	N	1	-	-
*Zantoxylum martinicensis* (Lam.) DC.	Rutaceae	A	N	1	-	-
*Guzmania monostrachya* (Sw.) Rusby	Bromeliaceae	Ep	N	+	-	-
*Microgramma piloselloides* L.	Polypodiaceae	Ep	N	+	-	-
**Companions species**						
*Cnemidaria horrida* (L.) K. Presl	Cyatheaceae	Ar	N	2	2	2
*Pytirogramma calomelanos* (L.) Link	Polypodiaceae	H	N	1	+	+
*Ipomoea tiliacea* (Willd.) Choisy	Convolvulaceae	Tr	N	+	2	2
*Mucuna urens* (L.) Fawc. & Rendle	Fabaceae	Tr	N	1	2	2
*Solanum torvum* Sw.	Solanaceae	Ar	N	1	1	1
*Mikania cordifolia* (L.) Willd.	Asteraceae	Tr	N	2	1	1
*Psychotria domingensis* Jacq.	Rubiaceae	Ar	N	-	2	1
*Pothomorphe peltata* (L.) Miquel	Piperaceae	Ar	N	-	2	2
*Tibouchina longifolia* (Vahl) Baill.	Melastomataceae	Ar	N	1	-	+
*Nepsera aquatica* (Aubl.) Naud.	Melastomataceae	Ar	N	1	-	+
*Syngonium podophyllum* Schott	Araceae	Tr	N	2	+	-
*Urera baccifera* (L.) Gaud.	Urticaceae	Ar	N	-	2	2
*Psychotria uliginosa* Sw.	Rubiaceae	Ar	N	-	2	2
*Coccocypselum herbaceum* Aubl.	Rubiaceae	H	N	+	-	-
*Piper adunculum* L.	Piperaceae	Ar	N	1	-	-
*Cissus verticillata* (L.) Nicholson & Farris	Vitaceae	Tr	N	1	-	-
*Neurolaena lobata* (L.) Cass.	Asteraceae	H	N	+	-	-
*Triunfetta semitriloba* Jacq.	Tiliaceae	H	N	1	-	-
*Clidemia umbellata* (Miller) L.O. Wms.	Melastomataceae	Ar	E	1	-	-
*Gleychenia bifida* (Willd.) Spreng.	Gleycheniaceae	H	N	1	-	-
*Lycopodium clavatum* L.	Lycopodiaceae	H	N	1	-	-
*Ichnanthus pallens* (Sw.) Munro	Poaceae	H	N	1	-	-
*Nephrolepis multiflora* (Roxb.) Jarret	Lomariopsidaceae	H	N	1	-	-
*Smilax domingensis* Willd.	Smilacaceae	Tr	N	+	-	-
*Mimosa pudica* L.	Mimosaceae	H	N	1	-	-
*Palicourea crocea* (Sw.) Schultes	Rubiaceae	Ar	N	1	-	-
*Urena lobata* L.	Malvaceae	Ar	N	1	-	-
*Hedychium coronarium* Koen.	Zingiberaceae	H	I	1	-	-
*Solanum jamaicense* Mill.	Solanaceae	Ar	N	1	-	-
*Entada gigas* (L.) Fawc. & Rendle	Fabaceae	Tr	N	+	-	-

Sites sampled: DR15—El Trece (eastern range) (19Q0489524/2092418). DR16—Dieciseis de Mitche (19Q0486735/2092513). DR17—Near Dieciseis de Mitche (19Q0486736/2092514). Tree = A; Shrub = Ar; Climber = Tr; Epiphyte = Ep; Herb = H; Native = N; Endemic = E; Introduced = I; Xn = Average height of the dominant species.
